# Oral reactive capillary hemangiomas induced by SHR-1210 in the treatment of non-small cell lung cancer: a case report and literature review

**DOI:** 10.1186/s12903-021-01901-9

**Published:** 2021-11-02

**Authors:** Jinhan Zhou, Qinghua Mao, Yining Li, Zhiyong Li, Hong He, Qianming Chen, Chuanxia Liu

**Affiliations:** Stomatology Hospital, School of Stomatology, Zhejiang University School of Medcine, Zhejiang Province Clinical Research Center for Oral Diseases, Key Laboratory of Oral Biomedical Rsearch of Zhejiang Province, Cancer Center of Zhejiang University, Hangzhou, 310006 China

**Keywords:** Reactive capillary hemangiomas, Oral, Anti-PD-1 antibody, Non-small cell lung cancer, Case report

## Abstract

**Background:**

Antibodies to PD-1 and PD-L1 have remarkably improved the overall survival of many patients with advanced solid tumors. SHR-1210 is an anti-PD-1 monoclonal antibody. Dermatologic reactive capillary hemangiomas (RCH) were the most common and unique drug-related AEs of SHR-1210, but rare on oral mucosa and gastrointestinal mucosa. Herein we report a case of RCH occurred in oral mucosa during the clinical trials of SHR-1210 in the treatment of non-small cell lung cancer.

**Case presentation:**

A male in his 60 s with a history of non-small cell lung cancer received injection of anti-PD-1 monoclonal antibodies SHR-1210. The patient developed drug-related RCH on skin after the first injection and began to have gingival hyperplasia one year after the first injection which gradually increased in size and affect eating and speaking. Anti-PD-1 treatments were continued. After periodontal treatment, two oral lesions and one skin lesion were surgically removed. Similar histological manifestation was found in all three lesions as reactive capillary hemangiomas. All lesions had a good prognosis without recurrence on oral mucosa within one year after surgery.

**Conclusions:**

Oral reactive capillary hemangiomas could be induced by SHR-1210 in the treatment of non-small cell lung cancer. Surgical resection is an effective treatment with a good prognosis.

## Background

Recently, breakthroughs in immunotherapy, including immune checkpoint blockade, are becoming a highly effective treatment of multiple malignancies refractory to conventional chemotherapies [1–3]. The programmed death 1 (PD-1) inhibitors inhibit the interaction between PD-1 and its ligand, PD-L1 and PD-L2, present on the surface of the tumor cells and immune cells in the tumor microenvironment, thereby activating immune responses toward cancer cells. Antibodies to PD-1 and PD-L1 have remarkably improved the overall survival of many patients with advanced solid tumors [4, 5]. Because of drug-mediated hyperactivation of the immune system, a spectrum of novel immune-related adverse events (irAEs) are put in front of oncologists. The most common irAEs involve thyroiditis, diarrhea, hepatitis, pneumonitis, and skin reactions. Dermatologic adverse events (DAEs) which include rash, pruritus, vitiligo, maculopapular, follicular, pustular, vesicular, acneiform, exfoliative lesions, etc., remain significant in 20–30% of patients undergoing treatment with PD-1 inhibitors (e.g. pembrolizumab and nivolumab) [6–8].

SHR-1210 is a selective, humanized, high-affinity IgG4-kappa mAb against PD-1. At present, it was under phase II clinical trial in esophageal cancer, non-small cell lung cancer, breast cancer, colon cancer and other solid tumors. Preliminary observation showed that SHR-1210 demonstrated a promising antitumor activity of SHR-1210 [9, 10]. Meanwhile, it was very important to focus on adverse events of SHR-1210. According to the researches, drug-related reactive capillary hemangiomas (RCH) was the most common and unique adverse reaction which was different from other anti-PD-1 antibodies. RCH occurred most often on the skin of face, scalp, neck, and chest, but rare on oral mucosa and gastrointestinal mucosa [11]. Herein we report a case of reactive capillary hemangiomas occurred in oral mucosa during the clinical trials of SHR-1210 in the treatment of non-small cell lung cancer.

## Case presentation

**Fig. 1:**
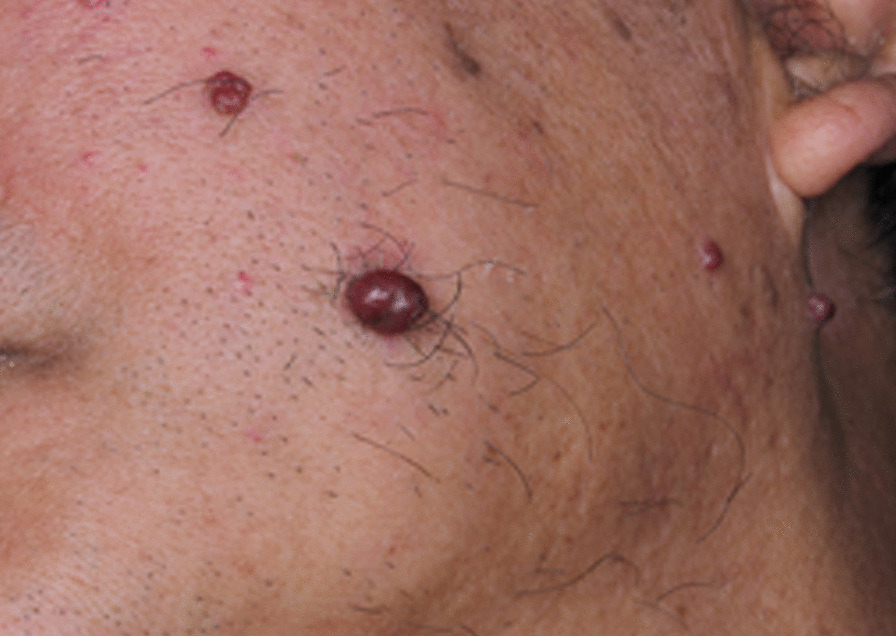
Reactive capillary hemangiomas (RCH) on the face, purple, dome-shaped, bright red papules

**Fig. 2:**
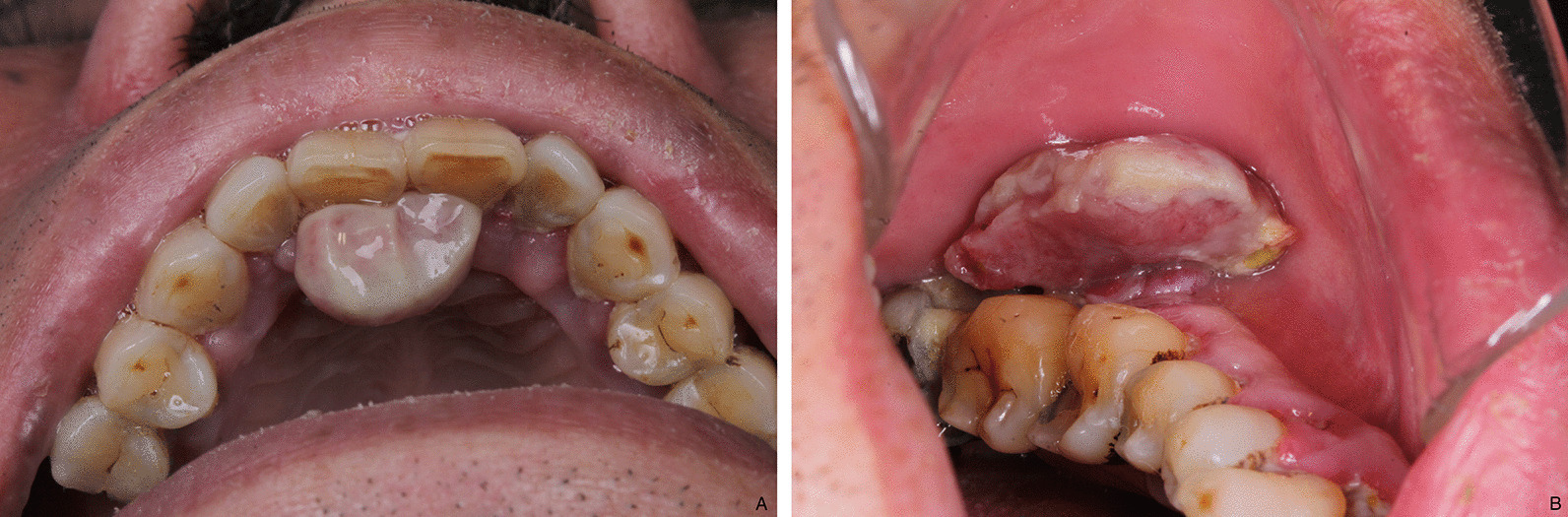
Gingival RCH, soft, pedunculated, no ulceration on the surface of mucous, easy to bleed when touched. **a** One lesion located on the palatal gingiva of the maxillary anterior teeth developed into 10 × 5 mm. **b** another lesion located on the buccal gingiva of the lower left posterior molar developed into 15 × 7 mm

**Fig. 3:**
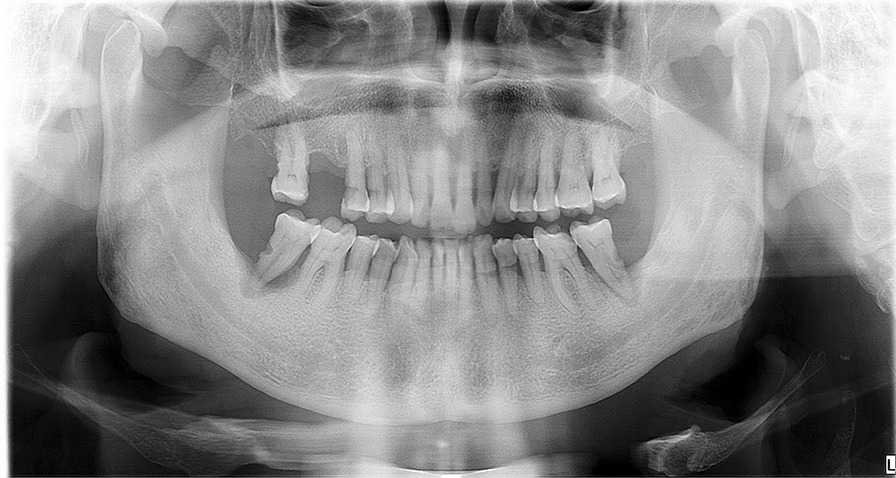
Panoramic radiograph showed no obvious bone resorption except periodontitis

A male in his 60 s with a history of non-small cell lung cancer was enrolled in a phase I/II trial for a humanized anti-PD-1 IgG4 antibody camrelizumab (SHR-1210). He was suffered from diabetes, hypertension, and hyperlipemia, treated with acarbose tablets, Adalat, Betaloc ZOK, Acertil, and fenofibrate capsules. After the first SHR-1210 injection, he began to have several, 1–3 mm in size, purple, dome-shaped, bright red papules on his face, chest, abdomen and hand (Fig. 1). SHR-1210 therapy was continued, and the lesions gradually increased in size and then regressed. One year after the first SHR-1210 injection, he began to have gingival hyperplasia and the lesions gradually increased in size. One lesion located on the palatal gingiva of the maxillary anterior teeth developed into 10 × 5 mm, another lesion located on the buccal gingiva of the lower left posterior molar developed into 15 × 7 mm, soft, pedunculated, no ulceration on the surface of mucosa, easy to bleed when touched (Fig. 2a, b). There was no obvious pain, but the lesions were easy to bleeding when eating and speaking. The oral hygiene was poor. Panoramic radiograph showed alveolar bone resorption resulted from periodontitis and 47 apical periodontitis but no specific bone destruction in gingival hyperplasia area (Fig. 3).

**Fig. 4:**
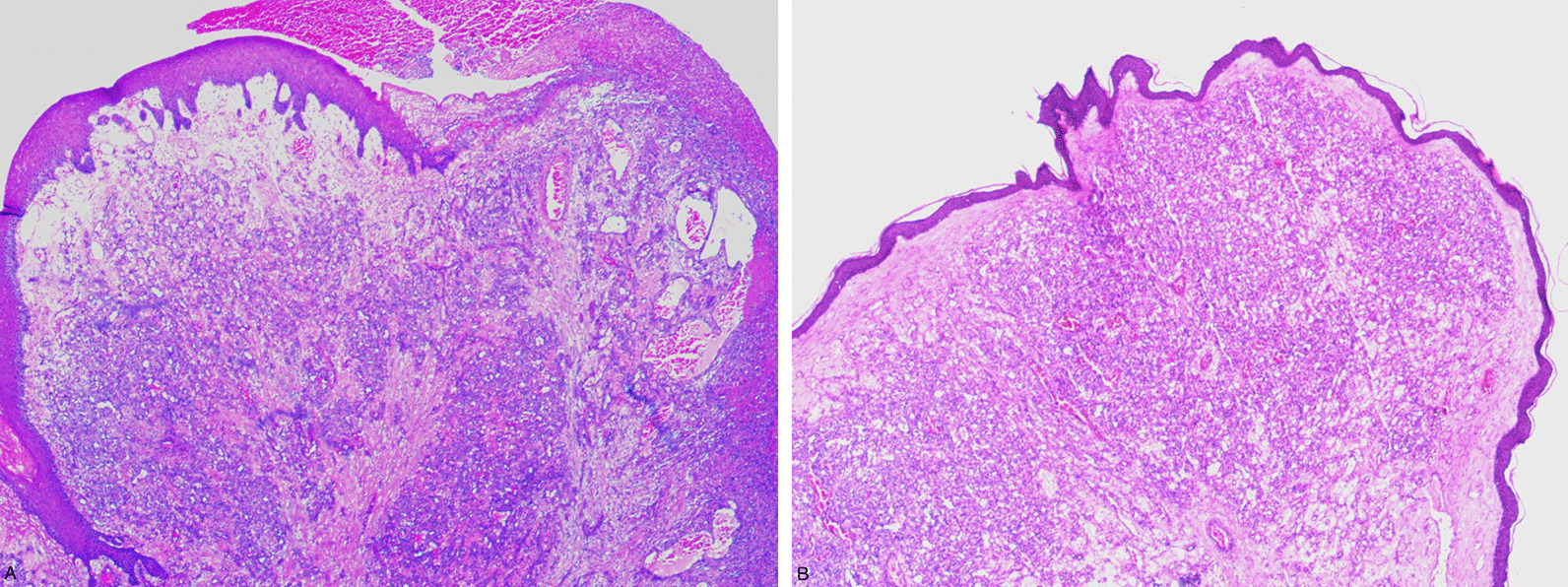
Histological manifestation of RCH. **a** Gingiva lesion showed epithelial erosion, lamina capillaries and vascular endothelial cells proliferate in a lobulated shape. **b** Skin lesion from the face showed proliferation of dermal capillaries and vascular endothelial cells in a lobulated shape

Conservative treatment such as periodontal and endodontic treatment to remove dental plaque, dental calculus, dental caries, and residual teeth with sharp tips were performed. However, the lesions in oral cavity remained and did not get any better. Then the two oral lesions and one lesion of the face were surgically removed. The histological manifestation of gingiva lesions was epithelial erosion, lamina capillaries and vascular endothelial cells proliferate in a lobulated shape (Fig. 4a). The histological manifestation of skin lesions of the face was the proliferation of dermal capillaries and vascular endothelial cells in a lobulated shape (Fig. 4b). The histology of these lesions combined with their clinical appearance was considered as reactive capillary hemangiomas related to SHR-1210. All lesions have a good prognosis without recurrence within one year after surgery.

## Discussion and conclusions

PD-1 expressed by activated T lymphocytes is a pivotal immune checkpoint receptor mediating immunosuppression once binding to the PD-1 ligands PD-L1 and PD-L2 expressed by tumor cells or stromal cells [5, 12]. The immune checkpoint blockade of PD-1 and PD-L1 pathway has emerged as one of the most potential therapeutic strategies in a variety of cancers, such as head and neck squamous cell carcinoma, non-small cell lung cancer, advanced esophageal squamous cell carcinoma, renal cell carcinoma, etc. [10, 11, 13–15]. Several monoclonal antibodies against PD-1 and PD-L1, such as nivolumab, cemiplimab, pembrolizumab, atezolizumab, etc., have been proved to be effective in the management of several malignancies [13, 14, 16]. However, their adverse reactions also attracted the attention of scholars while the immunosuppressive agents bring a surprise effect. In a normal adaptive immune response, immune checkpoint inhibition is in place to ensure that immune cells do not harm the host when responding to a foreign antigen. Interfering with this mechanism can cause immune-related adverse events directed against self-tissues. DAEs are some of the most frequently observed toxicities of immune-checkpoint inhibitor therapy. Rash, pruritus and vitiligo were found to be the most frequently reported DAEs, the incidence of nivolumab and pembrolizumab is 34% [17, 18]. However, serious dermatologic adverse events are rare, and usually do not need to stop treatment or drug reduction.

SHR-1210, a novel antibody against PD-1, has presented encouraging efficacy in patients with advanced gastric, advanced esophageal carcinoma, lung cancer, nasopharyngeal carcinoma, gastroesophageal junction cancer, etc. [9, 19–22]. Unlike other PD-1 inhibitors, dermatologic RCH was the most common and unique drug-related AEs of SHR-1210. The incidence rate was about 80% [9–11, 23]. Its mechanism was not clear, which may be related to the stress-induced vascular endothelial cell immune response. Prolonging the medication interval may reduce its occurrence and scope, and there was no effective preventive measure currently. The prognosis of dermatologic reactive capillary hemangiomas was good, most of them disappeared during and within 1–2 months after stopping SHR-1210. However, RCH rarely occurred in oral mucosa or gastrointestinal mucosa.

In our case, one year after the first SHR-1210 injection, he began to have gingival reactive capillary hemangiomas and the lesions gradually increased in size. The risk of hemoptysis and gastrointestinal bleeding cannot be ignored if RCH occurs in the mucosa of the respiratory tract and digestive tract. In this case, the patient received periodontal treatment in the first place, yet without any satisfying progress, which indicated these lesions were not inflammation hyperplasia stimulated by periodontitis or trauma. Because of the large size affecting chewing and speaking, and easy to rupture and bleed, the two gingival reactive capillary hemangiomas were surgically removed and have no recurrence within one year. In order to clarify the actual diagnosis of the neoplasm in oral cavity and skin, a pathological examination was performed, which showed a remarkable similarity in histopathological features of gingiva and skin lesions. Both of them showed the proliferation of capillaries and vascular endothelial cells in a lobulated shape. As far as our knowledge, this was the first time to compare the pathology of skin and gingiva at the same time. The pathology of RCH still remains a mystery but may be related to periodontal health. Differential diagnosis plays a critical role in these patients since epulis, trauma-related pyogenic granuloma and even tumor distant metastasis can demonstrate as similar neoplasm in oral cavity. We, therefore, highly recommend routine oral examination for patients with SHR-1210 treatment. Potential pathogenic factors such as dental plaque, dental calculus, and residual teeth with sharp tips are supposed to be removed to maintain good oral hygiene before the use of SHR-1210. Patients should sustain regular oral checkups until 1 year after immunotherapy to monitor any occurrence of neoplasm in oral cavity. After conservative treatment to exclude other diseases, surgical performance should be considered as a priority to remove RCH. The key contribution of this case is the foremost discovery of reactive capillary hemangiomas in oral cavity of SHR-1210 patients. The pathogenesis, prevention and treatment measures of oral RCH need to be further studied.

## Data Availability

The datasets used and/or analyzed during the current study are available from the corresponding author on reasonable request.

## Abbreviations

RCH: Reactive capillary hemangiomas
PD-1: Programmed death 1
PD-L1: Programmed cell death 1 ligand 1
irAEs: Immune-related adverse events
DAEs: Dermatologic adverse events
